# Supplementary material for: Fast myosin binding protein C knockout in skeletal muscle alters length-dependent activation and myofilament structure
Source: Commun Biol. 2024 May 27;7:648. doi: 10.1038/s42003-024-06265-8 (PMC11130249; doi:10.1038/s42003-024-06265-8)
Supplement: Supplementary file 2 — Supplementary Information [file 42003_2024_6265_MOESM2_ESM.pdf]

Supplementary Information for

Fast myosin binding protein C knockout in skeletal muscle alters length-dependent activation and myofilament structure.

Anthony L. Hessel[1*], Michel N. Kuehn[1], Seong-Won Han[1], Weikang Ma[2], Thomas C. Irving[2], Brent A. Momb[3], Taejeong Song[4], Sakthivel Sadayappan[4], Wolfgang A. Linke[1], Bradley M. Palmer[5*].

[1]Institute of Physiology II, University of Muenster; Muenster, Germany.

[2]BioCAT, Department of Biology, Illinois Institute of Technology; Chicago, USA.

[3]Department of Kinesiology, University of Massachusetts – Amherst; Amherst, MA, USA.

[4]Center for Cardiovascular Research, Division of Cardiovascular Health and Disease, Department of Internal Medicine, University of Cincinnati, Cincinnati, OH, USA

[5] Department of Molecular Physiology and Biophysics, University of Vermont; Burlington, VT, USA.

[*]Corresponding authors Anthony L. Hessel and Bradley M. Palmer

Email: anthony.hessel@uni-muenster.de, Bradley.palmer@uvm.edu

The PDF file includes:

Supplementary Figures 1-2

Supplementary Figures

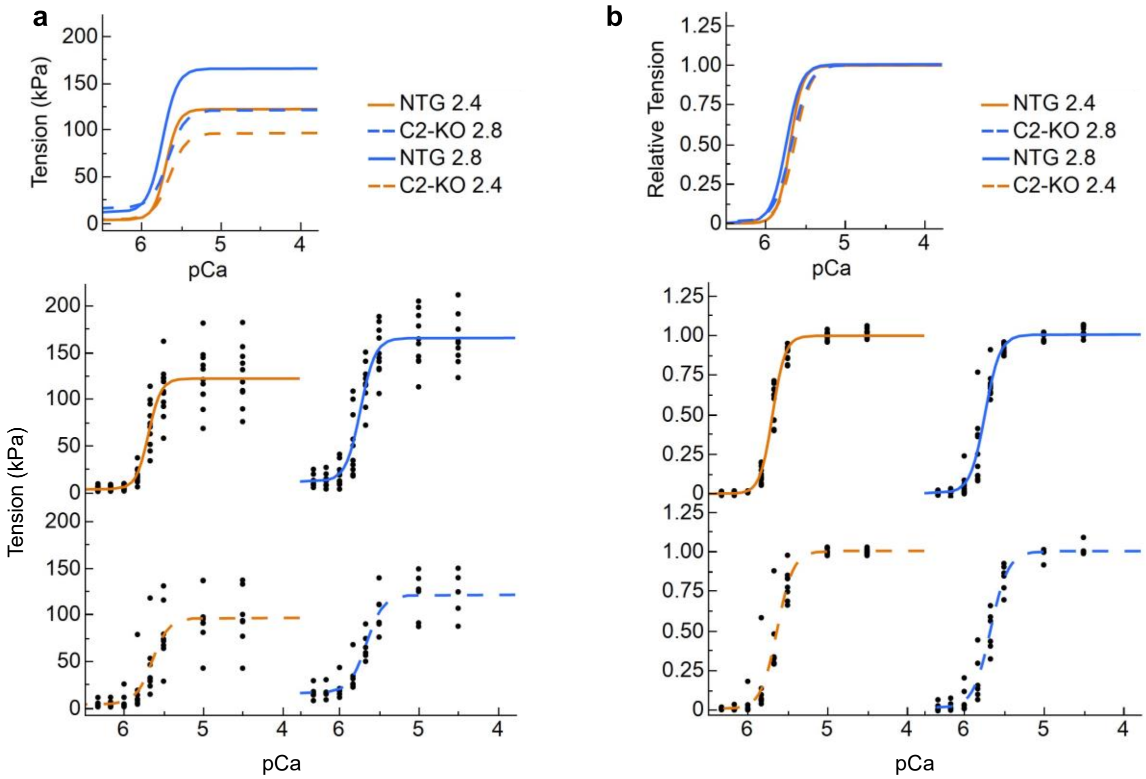

Supplementary Figure 1. Tension-pCa plots of NTG (solid lines) and C2$^{-/-}$ (dotted lines) at 2.4 (orange) and 2.8 (blue) µm SL. Also included are the individual data points for each condition in absolute (**a**) and relative (**b**) tension. Contains n = 6-10 per condition.

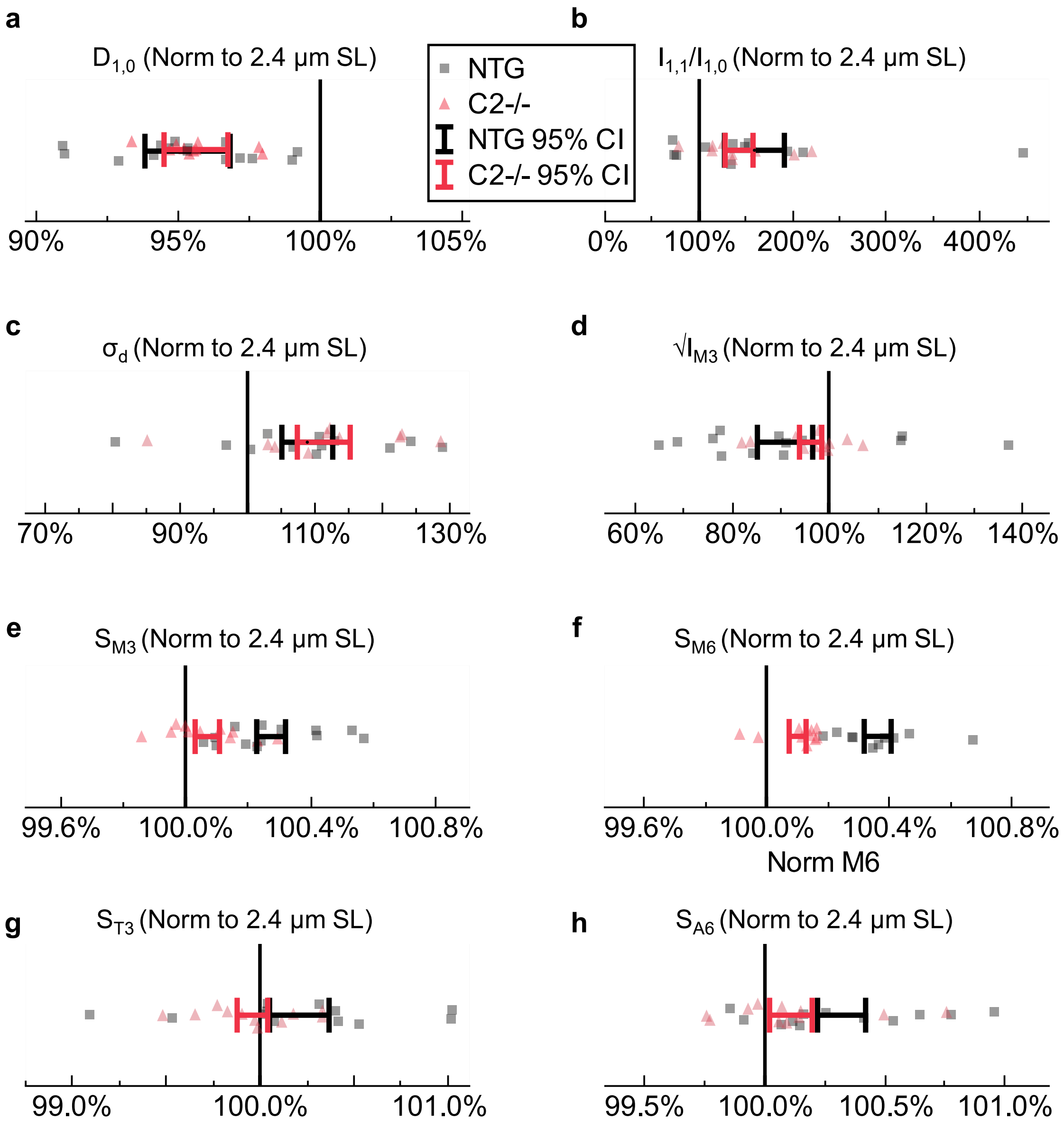

Supplementary Figure 2. Stretch-related relative change in structural features at 2.8 µm SL, normalized to 2.4 µm SL, for NTG (black squares) and $C2^{-/-}$ (red triangles) genotypes. (**a-h**) structural data presented as mean ± 95% confidence interval of the mean. 100% is the initial starting value for each sample at 2.4 µm SL.
