# Supplementary material for: Fast myosin binding protein C knockout in skeletal muscle alters length-dependent activation and myofilament structure
Source: Commun Biol. 2024 May 27;7:648. doi: 10.1038/s42003-024-06265-8 (PMC11130249; doi:10.1038/s42003-024-06265-8)
Supplement: Supplementary file 3 — Description of Additional Supplementary Files [file 42003_2024_6265_MOESM3_ESM.pdf]

Description of Additional Supplementary Files

File name: Supplementary Data 1
Description: The source data for all the figures and plots in the manuscript.
